# Barometric whole-body plethysmography investigating breed-specific variations in dogs

**DOI:** 10.3389/fvets.2026.1778874

**Published:** 2026-03-04

**Authors:** Charlotte Münch, Hannah Gareis, Simon Wiegrebe, Juliet Fleischer, Bianka Schulz

**Affiliations:** 1 Clinic of Small Animal Medicine, Center for Clinical Veterinary Medicine, Ludwig-Maximilians-University (LMU) Munich, Munich, Germany; 2 Statistical Consulting Unit StaBLab, Department of Statistics, Ludwig-Maximilians-University (LMU) Munich, Munich, Germany

**Keywords:** airway resistance, canine, obstructive respiratory disease, pulmonary function testing, respiratory tract

## Abstract

**Introduction:**

Barometric whole-body plethysmography (BWBP) is a non-invasive method for pulmonary function testing in dogs. A significant advantage over other techniques is the possibility to perform measurements in awake and unrestrained animals.

**Objective:**

To measure respiratory function parameters using BWBP in three different dog breeds and identify breed-specific differences and measurement variations.

**Materials and methods:**

Prospective comparative cross-sectional study including 41 clinically healthy dogs of the breeds Doberman pinscher (17), Parson Russell terrier (14), and French Bulldog (10), all at least 1 year of age. After system calibration, each dog underwent the measurement protocol, which consisted of a 5-min acclimatization period and followed by at least 15 min actual recording. Data were summarized using descriptive statistics and analyzed using separate linear models for each lung parameter with breed as a covariate, followed by multiplicity-adjusted pairwise breed comparisons. Additional pairwise tests assessed breed effects with adjustment for age or weight; statistical significance was set at *p* < 0.05.

**Results:**

Of 59 dogs recruited, 41 could be included in the final analysis. In the breed comparison, significant differences were identified for the parameters peak expiratory flow (PEF), peak inspiratory flow (PIF), expiratory flow at 50% of tidal volume (EF50), end-expiratory pause (EEP), and the ratio parameter Te/Ti. After adjusting for age and weight as covariates, significant differences among the three breeds persisted.

**Discussion:**

The findings support the assumption that breed-specific differences exist for BWBP parameters. However, future studies with larger sample sizes are required to establish reliable reference values, taking breed-specific differences into account.

## 1 Introduction

Barometric whole-body plethysmography (BWBP) is a non-invasive method for lung function testing and offers a clear advantage over conventional invasive procedures (1, 2). Animals can be examined awake and unrestrained, and the measurement only takes a few minutes, making it an attractive alternative to other pulmonary function tests, such as arterial blood gas analysis or measurement of lung compliance (3–5).

BWBP was first developed in human neonatology and later adapted for adults (6). The respiratory output produced by BWBP is designated as pseudo-flow, reflecting its indirect measurement, as the animals cannot breathe actively through a mouthpiece as adults humans do (7). During the measurement, the animal sits in an airtight plexiglass chamber which is equipped with sensitive sensors and connected to a computer software application that displays the lung function parameters in real-time. The measurement results are based on the pressure and volume changes that are recorded using a pneumotachograph and require trained staff for interpretation (2, 8). The method is based on the Boyle–Mariotte law, which relates chamber pressure to lung volume (9). In addition, the principle of isothermal gas compression and expansion plays an important, although subordinate, role in BWBP (10, 11).

In veterinary medicine, airway resistance cannot be quantified directly as specific airway resistance (sRaw) or airway resistance (Raw) in the same way as in human body plethysmography (12, 13). Instead, BWBP provides indirect indices that reflect changes in airflow limitation and are commonly interpreted as surrogates of increased airway resistance. In the present study, the authors therefore relied on established surrogate parameters, including Penh, the PEF/PIF ratio, PEF and PIF, Ti and Te, and ventilatory measures such as TV and MV. Collectively, these variables capture alterations in breathing pattern and flow dynamics that are consistent with increased resistive load, and they enable a functional assessment of obstructive respiratory changes despite the absence of a direct Raw/sRaw measurement (14).

At present, BWBP is primarily used in research and university settings, and its widespread use in clinical practice remains pending. Previous studies have primarily focused on evaluating and validating the overall reliability of the method (1, 15–17), as well as assessing the effects of provocation testing (15, 18) and nebulization (19), and the impact of obesity (20, 21). In addition, a few studies have examined lung function in brachycephalic breeds compared with a mixed-breed reference group of healthy dogs; however, breed-specific reference values for this measurement technique are still lacking.

Dog breeds can be categorized into three cranial types: mesocephalic, brachycephalic and dolichocephalic (22–24). These conformational groups are commonly based on cranial measurements, such as the cephalic index, S-index, and craniofacial angle (23–25), although recent literature suggests that the S-index provides greater accuracy (23). Consistent with these conformational distinctions, computed tomography-based computational fluid dynamics has demonstrated cephalic-type–dependent variation in upper-airway pressure and resistance distributions, with brachycephalic dogs showing distinct patterns compared with mesocephalic and dolichocephalic breeds, attributable to differences of the nasal apertures and soft palate (26).

Brachycephalic Obstructive Airway Syndrome (BOAS) is a well-documented pathological condition associated with the characteristic skull morphology of brachycephalic breeds such as Pugs and Bulldogs (27–29). Due to upper airway abnormalities, affected dogs may show respiratory noise on auscultation, dyspnea, exercise and heat intolerance and cyanosis (30–33). Progressive secondary changes of the soft palate and larynx can culminate in life-threatening laryngeal collapse (29). Because clinical signs are often misperceived as breed-specific norms, even by veterinarians (34), objective severity assessment is warranted, including functional grading schemes and quantitative indices such as the BWBP-derived BOAS-Index (35).

The primary objectives of this study were to characterize between-breed differences in BWBP-derived lung function parameters and to document measurement-related variability and practical limitations associated with BWBP testing. Outside BOAS-focused research, breed-related variation in BWBP parameters has been investigated only to a limited extent. Given evidence from computational fluid dynamics demonstrating cephalic-type–dependent differences in airway resistance and increased inspiratory pressures in brachycephalic dogs attributable to upper-airway obstruction (26, 36), we hypothesised that breed- and conformation-associated variation would also be reflected in BWBP-derived outcomes. By quantifying the extent of such variation, this work aims to inform whether breed- or conformation-specific BWBP reference ranges are required to support broader clinical application and interpretation in veterinary practice.

## 2 Materials and methods

### 2.1 Study population

The study was approved by the Ethics committee of the Centre for Clinical Veterinary Medicine of Ludwig-Maximilian-University (LMU) Munich (367-28-07-2023). Consent for all procedures was obtained from the respective owners of the dogs.

**Figure 1:**
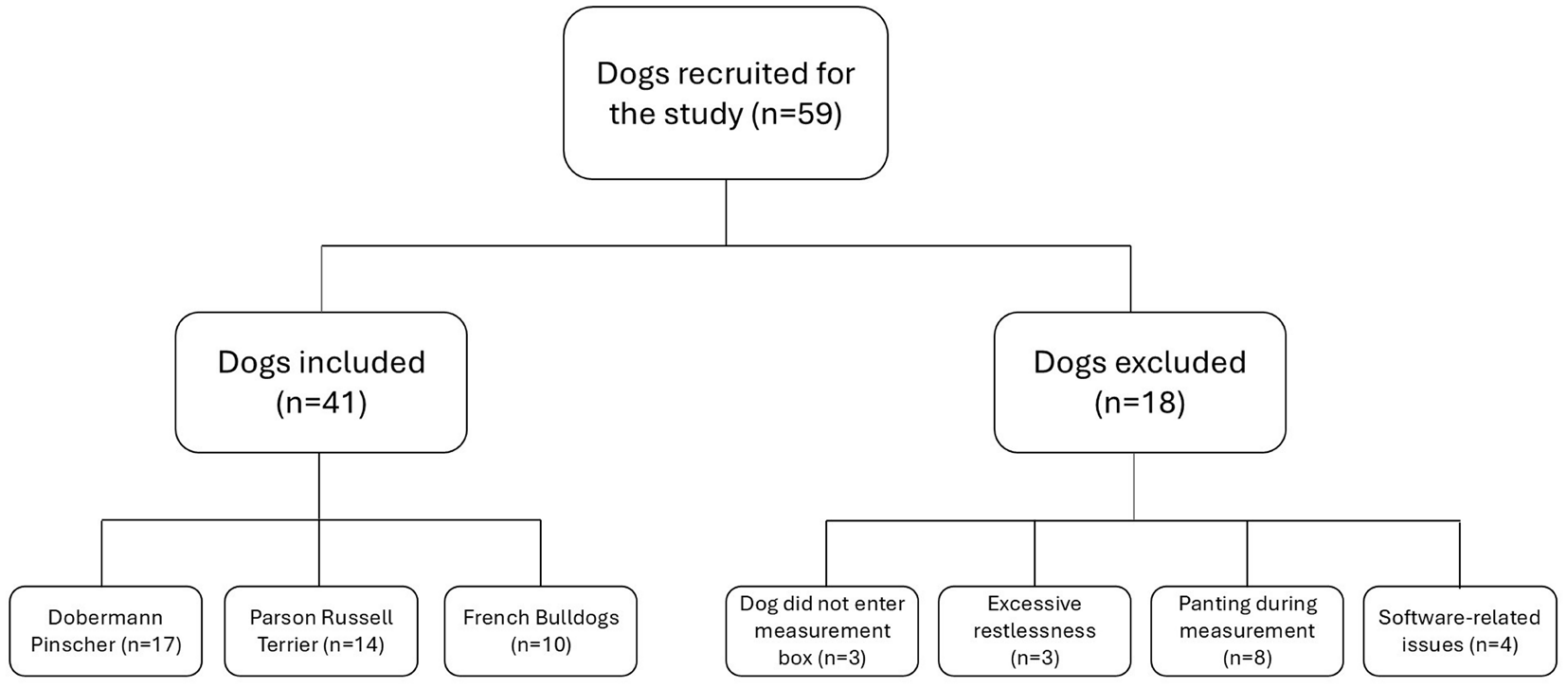
Flow chart of the study population. © C. Münch.

Clinically healthy client-owned dogs of the breeds Doberman pinscher (DP), Parson Russel terrier (PRT) and French bulldog (FB), aged 1 year or older, were included in the prospective study between December 2023 and July 2025. These breeds were selected to represent distinct cranial morphologies and body size, with FB representing a brachycephalic breed, PRT as a small mesocephalic breed and DP as an example for a large dolichocephalic breed. The selection also captured variation in thoracic conformation, including barrel-chested, keel-chested, and standard-chested phenotypes. At study enrolment, age, body weight, sex, neuter status, and body condition score were recorded for all dogs. Inclusion criteria consisted of the absence of clinically relevant findings on physical examination and no history of respiratory disease within the preceding 6 months. Dogs that exhibited signs of restlessness, such as persistent restlessness or panting during the measurements, had to be excluded (Figure 1). Additionally, for the FB group, eligibility was determined using the BOAS grading system by Liu et al. (37), with only dogs classified as grade 0 and 1 being included. Accordingly, FB in these groups exhibited no or only mild audible respiratory noise during auscultation prior to measurement, no or mild inspiratory effort, and showed no signs of clinically relevant respiratory compromise such as dyspnea, cyanosis or syncope. DPs were included only if no evidence of cardiac disease was identifiable at the time of enrolment based on physical examination and medical record. Where available, prior echocardiographic examinations were reviewed.

### 2.2 Study design

Before each client-owned dog underwent plethysmographic measurement, a standardized clinical examination was performed by the same veterinarian throughout the entire study period. Each dog was evaluated for a single measurement only. Dogs had not been trained for the plethysmographic chamber before measurement. No randomization was applied to chamber placement. Dogs entered the chamber voluntarily and were allowed to adopt a self-selected posture during the recording in accordance with the standardized measurement protocol.

### 2.3 Barometric whole-body plethysmography

Pulmonary function was measured using a large animal whole body plethysmograph (Buxco FinePoint Large Animal Whole Body Plethysmograph, Data Science International (DSI), New Brighton, Minnesota, USA) in a separate, quiet room within the small animal clinic, minimizing disruption from routine clinical activity and background noise. To ensure permanent ventilation of the patients in the airtight chamber, a bias flow (Buxco1 Multi-function Bias Flow, DSI, New Brighton, Minnesota, USA) was used. In addition, sieve pneumotachographs were attached to the chamber, allowing air flow in and out of the chamber, by a known air resistance. For measurement of the pressure changes in the chamber, a pressure transducer was connected to the plexiglass box and recording the flow rate “box flow.” The pressure transducer was also connected to a preamplifier (Buxco1QT Digital Preamplifier, DSI, New Brighton, Minnesota, USA) and the chamber signals were forwarded to a computer with an associated software program (Buxco1 FinePoint Large Animal Whole Body Plethysmograph, DSI, New Brighton, Minnesota, USA). This enabled real-time documentation of pressure changes in the chamber. The following parameters were obtained for every study participant: respiratory rate, tidal volume, minute volume, enhanced pause, pause, peak inspiratory flow, peak expiratory flow, inspiratory time, expiratory time, expiratory flow at end-tidal volume plus 50% tidal volume, end inspiratory pause, end expiratory pause and relaxation time. For the detailed explanation and the respective units, see Table 1: Summary of the BWBP-derived parameters, including their terminology, reported units, and a brief explanation.

**Table 1:** Summary of the BWBP-derived parameters, including their terminology, reported units, and a brief explanation.

Parameter	Term	Unit	Explanation
RR	Respiratory rate	BPM	Number of breaths per minute
TV	Tidal volume	mL	Air volume per breath
MV	Minute volume	mL/min	Total amount of air inhaled or exhaled in 1 min
Penh	Enhanced pause		An indicator of bronchoconstriction Penh = PEF/PIF × (Te − Tr)/Tr
PAU	Pause		Comparison of early versus late expiration times; indicator of bronchoconstriction PAU = (Te – Tr)/Tr
PIF	Peak inspiratory flow	mL/s	Highest inspiratory flow value during a breath
PEF	Peak expiratory flow	mL/s	Highest expiratory flow value during a breath
Ti	Inspiratory time	sec	Duration of inhalation
Te	Expiratory time	sec	Duration of exhalation
EF50	EF50	mL/s	Expiratory flow when 50% of the tidal volume has been exhaled
EIP	End-inspiratory pause	ms	Time interval between the end of inhalation and the start of exhalation
EEP	End-expiratory pause	ms	Time interval between the end of exhalation and the start of inhalation
Tr	Relaxation time	sec	Time required to go from maximum inhalation to maximum exhalation
PEF/EF50	PEF/EF50		Quotient of peak expiratory flow divided by expiratory flow at the point 50% of tidal volume is exhaled

**Figure 2:**
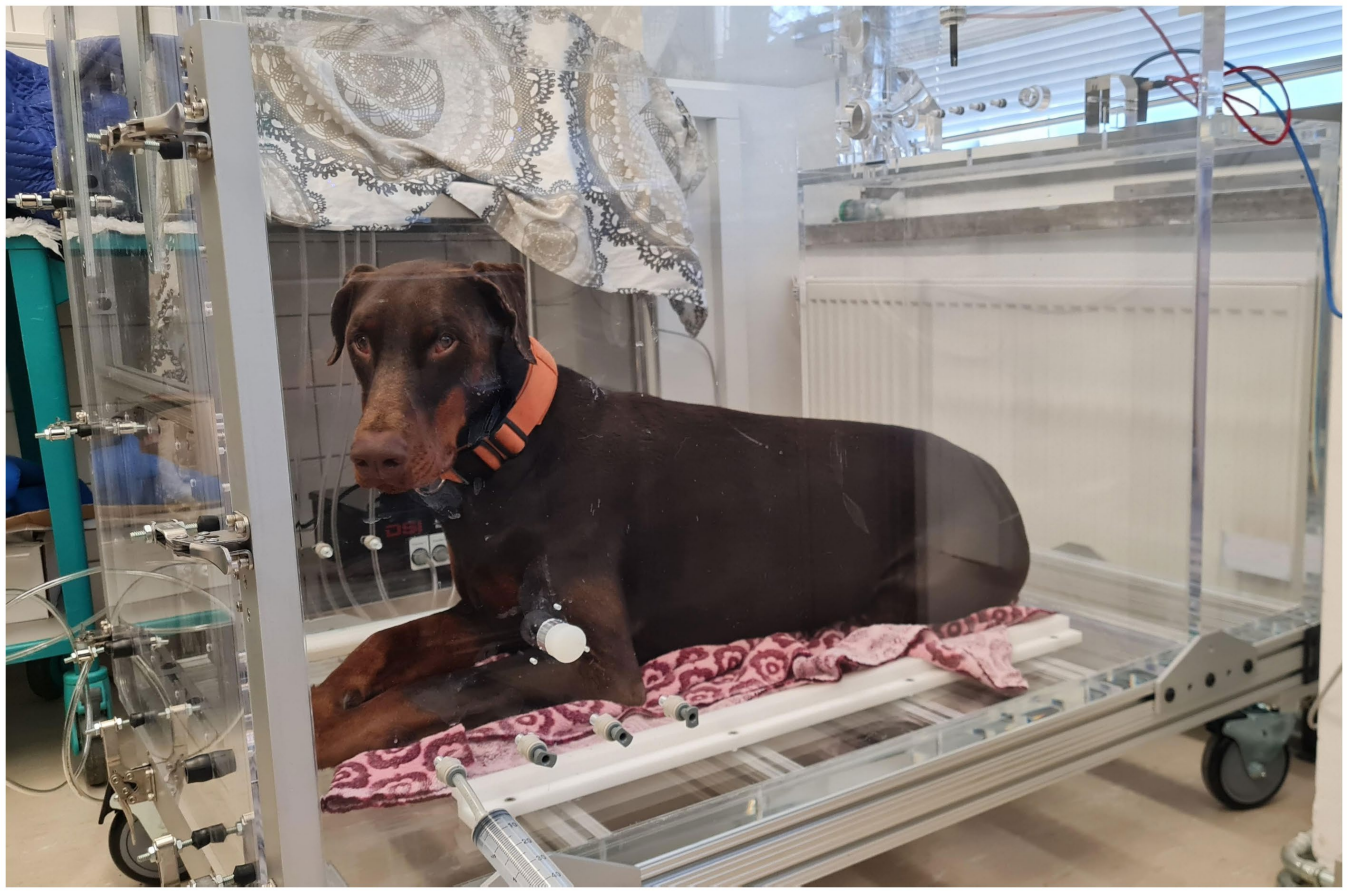
Dog in the BWBP measurement chamber. © C. Münch.

Prior to each measurement, system pressure calibration was performed by injecting 50 mL of room air into the sealed chamber. Each dog then entered the chamber voluntarily, unrestrained, awake, and without any sedation or premedication (Figure 2). Following a 5-min acclimatization period, the actual measuring period lasted at least 15 min, depending on how well the measurement was tolerated by the dog. The software automatically identified and excluded artefactual waveforms in real-time, including those caused by panting, sniffing, body movements or vocalization.

### 2.4 Statistical analysis

Statistical analysis was performed using R (R version 4.4.3 (2025-02-28), Copyright (C) 2025 The R Foundation for Statistical Computing). Data cleansing was performed by excluding the acclimatization period as well as measurement lines with a respiratory rate above 40/min. For each group, median age and body weight were summarized together with the corresponding minimum–maximum ranges, and the median body condition score was reported. Separate linear regression models were fitted for each lung parameter with breed as a covariate. To assess statistical differences in selected lung parameters, pairwise comparisons among the three breeds were conducted separately for each parameter. Multiple testing was accounted for using Tukey’s Honest Significant Difference (HSD) procedure, which controls the family-wise error rate. Additional pairwise comparisons under control of weight or age were performed to assess robustness of the estimated effects. Results were considered statistically significant at an adjusted *p*-value < 0.05.

## 3 Results

### 3.1 Study population

A total of 59 dogs were initially recruited for the study. Overall exclusion proportions were 16.7% (2/12) in FBs, 17.6% (3/17) in PRTs and 43.3% (13/30) in DPs. Exclusion was attributed to refusal to enter the measurement chamber voluntarily (*n* = 3), excessive restlessness or moving during measurement for more than 5 min (*n* = 3), panting (*n* = 8), or software-related problems (*n* = 4). Ultimately, 17 DP, 14 PRT and 10 FB were included in the final analysis. DP had a median age of 6.5 years (range 2.2–10.9) and a median body weight of 35.2 kg (range 21.7–49.0). The group included 19 males and 11 females, of which 18 were neutered and 12 intact. The median body condition score (BCS) was 5/9. PRT had a median age of 9.6 years (range 4.2–15.3) and a median body weight of 7.3 kg (range 6.5–9.7). The group comprised 12 females and 5 males, with 6 neutered and 11 intact individuals. The median BCS was 4.8/9. FB had a median age of 8.2 years (range 2.1–11.8) and a median body weight of 12.2 kg (range 9.0–17.8). This group included 4 females and 8 males, 8 neutered and 2 intact individuals, with the neuter status of 2 dogs unknown. The median BCS was 5/9.

### 3.2 Barometric whole-body plethysmography parameter

An overview of the median and interquartile range (IQR; Q1–Q3) estimates for each parameter, allowing direct comparison across breeds, is shown in Table 2. Comparative statistical results are summarized in Tables 3–5, and only statistically significant findings with *p*-values below 0.05 are presented here. The complete statistical outputs, including comparisons with *p*-values of 0.05 or greater, are provided in the Supplementary Tables S1–S3. Breed-specific differences were detected for several functional respiratory parameters. These included PIF, PEF, EF50, EEP and Te/Ti (Table 3). The pairwise tests with age and weight as covariates yielded results largely consistent with the breed comparisons. When controlling for weight, the parameters PEF/PIF and Te/Ti differed significantly (Table 4). In contrast, the analysis including age as a covariate revealed a greater number of significant effects. As shown in Table 5, significant differences among the three breeds were detected for the parameters PIF, PEF, Ti, Te, EF50, EEP, Te/Ti and PEF/EF50.

**Table 2:** Median (IQR; Q1–Q3) values for BWBP parameters for the three breeds [DP: Dobermann Pinscher (*n* = 18), FB: French Bulldog (*n* = 10), PRT: Parson Russell Terrier (*n* = 14)].

Parameter	DP	FB	PRT
RR	21.67 (16.53–24.24)	22.10 (17.15–25.99)	18.92 (15.08–20.07)
TV	372.55 (353.60–524.91)	130.54 (111.71–153.03)	102.85 (94.58–121.45)
MV	7,961.04 (6,578.06–10,818.60)	2,427.42 (1,923.83–3,980.35)	1,722.46 (1,402.22–2,289.25)
Penh	0.89 (0.72–1.65)	0.99 (0.67–1.45)	1.42 (1.05–1.90)
PAU	1.40 (1.06–2.12)	1.27 (0.85–1.83)	1.81 (1.51–2.35)
PIF	654.53 (528.29–750.83)	172.56 (145.82–350.98)	138.60 (130.68–180.12)
PEF	341.67 (254.03–543.06)	123.42 (106.24–269.75)	96.89 (81.17–113.79)
Ti	1.06 (0.98–1.26)	1.44 (1.12–1.48)	1.28 (1.14–1.54)
Te	1.93 (1.79–2.56)	1.72 (1.39–2.38)	2.38 (2.04–2.86)
EF50	251.72 (206.80–356.85)	106.07 (78.30–242.83)	64.15 (54.15–91.61)
EIP	29.16 (22.54–35.85)	45.04 (42.08–78.48)	39.45 (34.55–54.24)
EEP	214.52 (81.80–505.10)	329.69 (220.66–650.73)	837.12 (542.34–1,246.27)
TV/BW	13.54 (10.69–14.42)	10.96 (10.11–14.16)	14.51 (12.79–16.81)
MV/BW	261.07 (222.99–299.20)	212.25 (196.38–335.74)	225.19 (186.48–304.77)
PIF/BW	20.88 (17.10–22.43)	17.01 (13.15–27.89)	18.61 (17.30–23.20)
PEF/BW	11.28 (8.94–15.09)	10.48 (9.91–23.00)	12.83 (11.51–16.18)
PEF/PIF	0.57 (0.47–0.82)	0.76 (0.66–0.88)	0.68 (0.63–0.72)
Te/Ti	1.90 (1.81–2.06)	1.40 (1.09–1.78)	1.92 (1.74–2.04)
PEF/EF50	1.38 (1.28–1.46)	1.31 (1.18–1.39)	1.48 (1.36–1.59)

**Table 3:** Pairwise interbreed comparison between the three breeds [DP: Dobermann Pinscher (*n* = 18), FB: French Bulldog (*n* = 10), PRT: Parson Russell Terrier (*n* = 14)].

Parameter	Breed comparison	Estimate	Standard error	*p*-value
PIF	FB–DP	−429.79	66.29	<0.001
PIF	PRT–DP	−525.37	60.03	<0.001
PEF	FB–DP	−239.93	97.82	0.048
PEF	PRT–DP	−358.42	88.58	<0.001
EF50	PRT–DP	−246.74	54.40	<0.001
EEP	PRT–DP	632.48	209.70	0.012
EEP	PRT–FB	603.44	240.57	0.042
Te/Ti	FB–DP	−0.45	0.14	0.009
Te/Ti	PRT–FB	0.44	0.15	0.014

The table reports the estimates and standard errors for comparisons with *p*-values < 0.05. Complete results, including non-significant findings, are provided in Supplementary Table S1.

**Table 4:** Pairwise interbreed comparison between DP, FB and PR the three breeds [DP: Dobermann Pinscher (*n* = 18), FB: French Bulldog (*n* = 10), PRT: Parson Russell Terrier (*n* = 14)], with body weight included as a covariate.

Parameter	Breed comparison	Estimate	Standard error	*p*-value
PEF/PIF	FB–DP	0.54	0.17	0.008
PEF/PIF	PRT–DP	0.49	0.19	0.042
Te/Ti	FB–DP	−0.74	0.28	0.031

The table reports the estimates and standard errors for comparisons with *p*-values < 0.05. Complete results, including non-significant findings, are provided in Supplementary Table S2.

**Table 5:** Pairwise interbreed comparison between the three breeds [DP: Dobermann Pinscher (*n* = 18), FB: French Bulldog (*n* = 10), PRT: Parson Russell Terrier (*n* = 14)], with age included as a covariate.

Parameter	Breed comparison	Estimate	Standard error	*p*-value
PIF	FB–DP	−426.52	68.75	<0.001
PIF	PRT–DP	−519.49	66.36	<0.001
PEF	FB–DP	−251.91	101.11	0.045
PEF	PRT–DP	−379.89	97.59	0.001
Ti	PRT–DP	0.30	0.12	0.045
Te	PRT–DP	0.65	0.26	0.043
Te	PRT–FB	0.77	0.28	0.020
EF50	FB–DP	−152.68	61.98	0.048
EF50	PRT–DP	−262.60	59.83	0.000
EEP	PRT–DP	751.72	226.76	0.006
EEP	PRT–FB	656.18	241.69	0.027
Te/Ti	FB–DP	−0.39	0.14	0.024
Te/Ti	PRT–FB	0.48	0.15	0.006
PEF/EF50	PRT–FB	0.24	0.09	0.041

The table reports the estimates and standard errors for comparisons with *p*-values < 0.05. Complete results, including non-significant findings, are provided in Supplementary Table S3.

## 4 Discussion

This study evaluated BWBP as a non-invasive approach to respiratory functional assessment across clinically healthy dogs representing distinct skull morphologies, body sizes, and thoracic conformations. The findings indicate that BWBP-derived outcomes vary systematically between breeds, with significant differences across multiple flow- and timing-related indices. Covariate analyses further showed that both body weight and age contributed to BWBP-derived variability, with age demonstrating broader associations than weight. From a feasibility perspective, breed-dependent acceptance of the procedure affected data completeness, as reflected by a higher exclusion rate in DP, underscoring that both biological and measurement-related sources of variability must be considered when interpreting BWBP-derived results and when contemplating conformation- or breed-specific interpretative frameworks.

In the present study, Te/Ti was significantly reduced in FBs both in the direct breed comparison and remained significant after adjustment for age and body weight as covariates. This indicates a relative predominance of inspiratory time and may result from a shortened expiratory phase prolonged inspiration, or both, and should therefore be interpreted alongside airflow indices and clinical signs. DPs showed a significantly lower PEF/PIF ratio than both FBs and PRTs in the direct breed comparison with body weight included as a covariate. After adjusting for age, DPs also showed significantly longer Te compared to FBs and PRTs, as well as significantly longer Ti than PRTs. Reduced Ti and Te typically indicate an overall shortening of the respiratory cycle consistent with increased respiratory drive like tachypnoea or panting. In dogs with BOAS, decreased Ti and Te may represent compensatory increases in breathing frequency secondary to elevated upper-airway resistive load and impaired thermoregulation and should be interpreted alongside airflow indices and clinical signs. In one study comparing BOAS-negative FBs defined as grade 0 and 1 with non-brachycephalic dogs the brachycephalic group showed significantly lower Te and Ti values as well as higher PEF/PIF ratios (35). Similar findings were reported in bulldogs and Boston terriers, when compared with healthy non-brachycephalic controls (38). A reduced Te/Ti ratio likely reflects prolonged inspiratory effort in response to increased upper airway resistance in brachycephalic dogs, resulting in a relatively longer Ti than Te (39). Overall, these findings are consistent with the present findings in FBs.

In this study, PIF and PEF were significantly higher in DPs compared to FBs or PRTs. In contrast, the body-weight-normalized indices PIF/BW and PEF/BW did not reach statistical significance, a finding likely attributable to the substantial variation in body weight within the study population. When body weight was included as a covariate, DPs exhibited significantly lower PEF/PIF ratios compared to both other breeds. This may indicate a relative predominance of inspiratory over expiratory peak flow and may reflect higher PIF, lower PEF, or a combination of both. In the absence of clinical evidence of airway obstruction in this group, the physiological basis of this finding remains unclear, and no direct inference regarding skull morphology can be drawn from the present data. One potential explanation is that comparatively low upper-airway resistance in dolichocephalic dogs may facilitate higher inspiratory peak flows. Nevertheless, this is merely a hypothesis and lacks evidence in current literature.

FBs showed a reduced EEP compared with the other breeds. They also exhibited a decreased Te/Ti ratio compared to the remaining groups, and their PEF/EF50 ratio was lower than that of the PRTs. Taken together, these patterns are compatible with altered airflow dynamics consistent with upper-airway obstruction and align with the pathological changes’ characteristic of BOAS, although only grade 0 and 1 FB were included in the present study (35, 38). Mid-expiratory flow (MEF) analysis was not included in the present study; however, Tomlinson et al. (40) have shown that BWBP can capture BOAS-related differences in airflow dynamics by assessing MEF at four points throughout the expiration.

Surprisingly, no significant breed-specific differences were detected among the study participants for the parameters Penh and PAU. Although these parameters are commonly reported as surrogate indices associated with bronchoconstriction or altered airway mechanics in plethysmographic recordings (14), increases in brachycephalic dogs would not necessarily reflect lower-airway bronchoconstriction. Instead, given the increased upper-airway resistance and altered breathing pattern associated with brachycephalic conformation, higher values might be anticipated if these derived indices are sensitive to upper-airway obstruction and changes in respiratory effort. Additionally, the calculated parameter PEF/EF50 has also been described as an indicator for bronchoconstriction (37). In this study, significantly different PEF/EF50 values were found in the pairwise comparisons between FBs and PRTs, when age was included as a covariate, with PRT showing significantly higher values. The parameter Penh has been discussed controversially in literature. Some studies in dogs and cats revealed Penh as a valid indicator of bronchoconstriction, both with (15, 18) and without (5, 14, 41, 42) bronchoprovocation testing. However, other publications described Penh as less sensitive and less indicative of bronchoconstriction (17). Experience and comparative data regarding Penh-based assessment of upper airway obstruction are limited in the literature. The absence of breed differences for Penh in the present study may be explained by the limited range of BOAS grades among the enrolled FBs. Inclusion of FBs across a wider BOAS severity spectrum could have permitted a more detailed evaluation of the relationship between BOAS severity and BWBP-derived indices of airflow limitation such as Penh. However, previous studies did not evaluate Penh in brachycephalic dogs, instead establishing a BWBP-derived BOAS index based on other airflow-related parameters (43, 44).

No significant breed differences could be demonstrated for TV/BW or MV/BW in our study, although a trend was observed in MV/BW. Similar findings have been reported previously, when authors suspected an influence of BCS on lung function, suggesting that changes in thoracic musculature and geometry may affect lung volume. However, they attributed the lack of detectable differences to the uniform and lean study population, comparable to the dogs included in our study (45). Another study involving BOAS-negative FBs found no significant differences in MV/BW compared with healthy controls (35). Evidence from both human (46, 47) and canine studies (20) indicates that obesity can influence and/or predispose individuals to impaired lung function.

So far, evidence regarding age-related effects on pulmonary function parameters in veterinary medicine is limited. FBs showed significantly lower Te/Ti compared to both breeds and significantly reduced PEF/EF50 compared to PRT. In PRTs, we detected a lower RR, prolonged Te and increased EEP compared with both other breeds. PRTs also represented the group with the highest median age. Whether this reflects a true association between age and breed, or a bias of the study population due to younger age of the other groups, remains unclear. The significant increases in PIF, PEF and EF50, as well as the prolonged Ti in DPs, may likewise be attributable to age-related pulmonary changes, as outlined above. However, direct evidence for this relationship is lacking in veterinary literature. In juvenile cats, age correlated with TV, MV, PIF, and PEF between 3 and 8 months of age, with no further changes documented thereafter, consistent with attainment of pulmonary maturity (3). Because all enrolled animals in the present study were 1 year or older, a direct comparison with juvenile cohorts and maturation-related changes is not possible. Human data indicate that aging reduces lung elastic recoil and chest-wall compliance, producing airflow limitation with declines in forced expiratory volume in one second and increases in residual volume and functional residual capacity consistent with air trapping and hyperinflation, while total lung capacity is largely preserved (48, 49). Aging is also associated with reduced respiratory-muscle strength, with maximal inspiratory pressure and maximal expiratory pressure declining with age (50). Taken together, the age-related changes in pulmonary function reported here, including the aforementioned parameters, are physiologically plausible and may reflect the combined effects of reduced chest-wall compliance and diminished expiratory muscle performance. As noted above, brachycephalic dogs also show altered timing indices (35), most notably a lower Te/Ti driven by prolonged inspiratory effort, so age related shifts in Te may compound these breed related ventilatory constraints. These observations are in line with the results of our study.

The results of the pairwise testing suggest that weight and age may act as a mediator, rather than a collider or confounder. Further research is needed to confirm this finding and to explore the mechanisms by which weight affects the relationship between the other variables.

Thoracic conformation and dimensions may influence canine respiratory mechanics, particularly compliance-related endpoints. In clinically normal dogs, static respiratory compliance has been shown to differ between narrow- and broad-chested individuals, supporting an effect of chest wall geometry on overall respiratory system distensibility (51). Similarly, thorax length has been reported to be positively associated with static compliance in anesthetized brachycephalic dogs, indicating that thoracic dimensions can contribute to functional variation (52). In contrast, fluoroscopic measures of thoracic and lung area changes during quiet breathing were correlated with body weight but not thoracic conformation in healthy dogs, suggesting that some expansion-based metrics may be driven predominantly by size rather than categorical chest shape (45).

In general, the measurement method used in this study was well tolerated by most dogs, and no measurements had to be discontinued due to respiratory distress. However, a higher exclusion rate was observed in DPs compared to the other two breeds. This may be attributable to the chamber being proportionally smaller relative to the body size of DPs compared with the other two breeds and the consequently lower air volume per body surface area. By contrast, persistent panting was consistently accompanied by a progressive increase in chamber temperature and relative humidity. In these instances, recordings were discontinued at the examiner’s discretion to mitigate distress and to avoid further deterioration of measurement conditions. To our knowledge, no studies demonstrate increased stress sensitivity in DPs compared to other breeds. Two PRTs were excluded from the analysis due to software-related issues. The authors assume this was related to the dogs’ low body weight. Previous studies have described this measurement method as being well tolerated, with strong supporting evidence particularly in cats (7, 18, 53–55). In studies where dogs underwent habituation and chamber training beforehand, the method was also well tolerated and did not induce obvious stress reactions (15, 56). In the present study, chamber training was intentionally omitted to reflect typical clinical practice conditions. However, other studies have reported that the measurement procedure can lead to restlessness, panting, excitement, excessive movement, or vocalization (15, 35, 43). Each study defined different exclusion criteria, for example, continuous restlessness during the first 5 min and persistent intolerance after a repeated attempt once the dog had calmed down (35, 43). In some studies, stress reactions were probably not observed, because the dogs were sedated for additional procedures (15, 55). However, sedation should not be considered a standard procedure for BWBP, as it is considered a non-invasive procedure and should only be applied when required for further clinical purposes, such as subsequent diagnostic imaging or therapeutic procedures. When comparing diagnostics between species, the measurement method seems generally better tolerated in cats than in dogs (15).

The main limitation of this study is the relatively small sample size, which results in limited statistical power. Additionally, no a-priori sample size calculation was performed prior to study initiation. As further limitation, an acclimatization period of 5 min may also have been insufficient, as some dogs might only become familiar with the environment after a longer acclimatization interval. However, this interpretation remains speculative and should be evaluated in subsequent studies. Moreover, the study population was specifically selected and included only three breeds, thereby restricting the generalizability of the findings. A separate external control group was not included because the study design focused on characterizing breed-specific BWBP patterns in clinically healthy dogs, and an additional mixed-breed control cohort, inevitably heterogeneous in conformation, could introduce confounding and potentially bias between-breed comparisons. Additionally, the motivation to participate in this study influenced the study population. Only client-owned dogs were enrolled, with owners volunteering to participate and without prior respiratory diagnostic work-up. Recruitment was particularly challenging for FBs, as many owners and contacted breeders declined participation, likely due to concern that their dogs might be affected by BOAS. In contrast, numerous owners of DP and PRTs proactively expressed interest in participating in order to have their dogs’ lung function evaluated as a preventive measure. Notably, breeder participation was successfully encouraged for PRTs, which may have influenced the study population and therefore the study outcomes.

Future studies should aim to include a larger and more heterogeneous population encompassing a broader range of breeds and a higher number of subjects. In addition, establishing breed- and conformation-specific reference intervals should be considered to support clinical interpretation of BWBP-derived parameters at the individual-patient level and to improve discrimination between physiological breed-related variation and clinically relevant respiratory dysfunction. If brachycephalic dogs are included, individuals with a range of BOAS grades should also be represented. Repeated measurements on different days could be considered to assess potential habituation effects; however, this approach may not be practical under routine clinical setting.

Overall, this prospective study demonstrates that BWBP-derived outcomes in clinically healthy client-owned dogs differ between breeds representing distinct skull morphologies, body sizes, and thoracic conformations. Several flow- and timing-related indices showed significant differences between breeds, and both age and body weight contributed to variability in BWBP-derived parameters. Feasibility constraints also affected data completeness, as indicated by breed-dependent acceptance and a higher exclusion rate in DP. These findings underscore that BWBP results cannot be interpreted independently of conformation, patient-related covariates, and procedural factors, and they provide a framework for cautious clinical use of BWBP when comparing individuals across breeds. Accordingly, clinical interpretation is likely to benefit from stratified benchmarks that reflect this conformation- and covariate-related variability.

## Data Availability

The original contributions presented in the study are included in the article/Supplementary material, further inquiries can be directed to the corresponding author.
