# Supplementary material for: Barometric whole-body plethysmography investigating breed-specific variations in dogs
Source: Front Vet Sci. 2026 Mar 4;13:1778874. doi: 10.3389/fvets.2026.1778874 (PMC12995626; doi:10.3389/fvets.2026.1778874)
Supplement: Supplementary file 1 [file Table_1.DOCX]

Supplementary Material

# Supplementary Tables

**Supplementary Table 1.** Pairwise interbreed comparison between the three breeds (DP: Dobermann Pinscher, FB: French Bulldog, PRT: Parson Russell Terrier). The table reports the estimates, standard errors and the p-values.

| **Parameter** | **Breed Comparison** | **Estimate** | **Standard Error** | **p-value** |
| --- | --- | --- | --- | --- |
| **f** | FB - DP | 0.53 | 2.06 | 0.964 |
| **f** | PRT - DP | -3.50 | 1.86 | 0.158 |
| **f** | PRT - FB | -4.03 | 2.14 | 0.156 |
| **Penh** | FB - DP | -0.16 | 0.31 | 0.869 |
| **Penh** | PRT - DP | 0.33 | 0.28 | 0.481 |
| **Penh** | PRT - FB | 0.48 | 0.32 | 0.301 |
| **PAU** | FB - DP | -0.30 | 0.28 | 0.526 |
| **PAU** | PRT - DP | 0.24 | 0.25 | 0.607 |
| **PAU** | PRT - FB | 0.54 | 0.29 | 0.158 |
| **PIF** | FB - DP | -429.79 | 66.29 | 0.000 |
| **PIF** | PRT - DP | -525.37 | 60.03 | 0.000 |
| **PIF** | PRT - FB | -95.57 | 68.87 | 0.356 |
| **PEF** | FB - DP | -239.93 | 97.82 | 0.048 |
| **PEF** | PRT - DP | -358.42 | 88.58 | 0.001 |
| **PEF** | PRT - FB | -118.49 | 101.62 | 0.480 |
| **Ti** | FB - DP | 0.24 | 0.12 | 0.130 |
| **Ti** | PRT - DP | 0.25 | 0.11 | 0.070 |
| **Ti** | PRT - FB | 0.01 | 0.13 | 0.996 |
| **Te** | FB - DP | -0.25 | 0.27 | 0.635 |
| **Te** | PRT - DP | 0.44 | 0.25 | 0.193 |
| **Te** | PRT - FB | 0.69 | 0.28 | 0.052 |
| **EF50** | FB - DP | -143.83 | 60.07 | 0.055 |
| **EF50** | PRT - DP | -246.74 | 54.40 | 0.000 |
| **EF50** | PRT - FB | -102.91 | 62.41 | 0.237 |
| **EIP** | FB - DP | 24.56 | 11.37 | 0.091 |
| **EIP** | PRT - DP | 6.52 | 10.30 | 0.803 |
| **EIP** | PRT - FB | -18.05 | 11.81 | 0.289 |
| **EEP** | FB - DP | 29.04 | 231.56 | 0.991 |
| **EEP** | PRT - DP | 632.48 | 209.70 | 0.012 |
| **EEP** | PRT - FB | 603.44 | 240.57 | 0.042 |
| **TV/BW** | FB - DP | 1.44 | 1.96 | 0.745 |
| **TV/BW** | PRT - DP | 2.44 | 1.78 | 0.363 |
| **TV/BW** | PRT - FB | 1.00 | 2.04 | 0.875 |
| **MV/BW** | FB - DP | 31.99 | 43.15 | 0.740 |
| **MV/BW** | PRT - DP | 5.27 | 39.07 | 0.990 |
| **MV/BW** | PRT - FB | -26.72 | 44.83 | 0.823 |
| **PIF/BW** | FB - DP | -0.67 | 3.28 | 0.977 |
| **PIF/BW** | PRT - DP | 2.07 | 2.97 | 0.767 |
| **PIF/BW** | PRT - FB | 2.73 | 3.41 | 0.703 |
| **PEF/BW** | FB - DP | 3.24 | 3.62 | 0.646 |
| **PEF/BW** | PRT - DP | 2.08 | 3.28 | 0.803 |
| **PEF/BW** | PRT - FB | -1.17 | 3.76 | 0.948 |
| **PEF/PIF** | FB - DP | 0.18 | 0.09 | 0.119 |
| **PEF/PIF** | PRT - DP | 0.05 | 0.08 | 0.820 |
| **PEF/PIF** | PRT - FB | -0.13 | 0.09 | 0.336 |
| **Te/Ti** | FB - DP | -0.45 | 0.14 | 0.009 |
| **Te/Ti** | PRT - DP | -0.01 | 0.13 | 0.998 |
| **Te/Ti** | PRT - FB | 0.44 | 0.15 | 0.014 |
| **PEF/EF50** | FB - DP | -0.13 | 0.09 | 0.334 |
| **PEF/EF50** | PRT - DP | 0.10 | 0.08 | 0.462 |
| **PEF/EF50** | PRT - FB | 0.23 | 0.09 | 0.052 |

**Supplementary Table 2.** Pairwise interbreed comparison between DP, FB and PR the three breeds (DP: Dobermann Pinscher, FB: French Bulldog, PRT: Parson Russell Terrier), with body weight included as a covariate. The table reports the estimates, standard errors and p-values.

| **Parameter** | **Breed Comparison** | **Estimate** | **Standard Error** | **p-value** |
| --- | --- | --- | --- | --- |
| **f** | FB - DP | 6.23 | 4.03 | 0.263 |
| **f** | PRT - DP | 3.59 | 4.71 | 0.712 |
| **f** | PRT - FB | -2.64 | 2.26 | 0.457 |
| **Penh** | FB - DP | 0.10 | 0.62 | 0.986 |
| **Penh** | PRT - DP | 0.64 | 0.73 | 0.640 |
| **Penh** | PRT - FB | 0.54 | 0.35 | 0.262 |
| **PAU** | FB - DP | -0.44 | 0.56 | 0.697 |
| **PAU** | PRT - DP | 0.07 | 0.65 | 0.994 |
| **PAU** | PRT - FB | 0.51 | 0.31 | 0.236 |
| **PIF** | FB - DP | -76.43 | 116.49 | 0.777 |
| **PIF** | PRT - DP | -86.02 | 136.07 | 0.791 |
| **PIF** | PRT - FB | -9.59 | 65.27 | 0.987 |
| **PEF** | FB - DP | 227.90 | 177.36 | 0.392 |
| **PEF** | PRT - DP | 223.25 | 207.19 | 0.513 |
| **PEF** | PRT - FB | -4.65 | 99.39 | 0.999 |
| **Ti** | FB - DP | 0.04 | 0.24 | 0.981 |
| **Ti** | PRT - DP | 0.01 | 0.29 | 1.000 |
| **Ti** | PRT - FB | -0.04 | 0.14 | 0.956 |
| **Te** | FB - DP | -0.98 | 0.53 | 0.161 |
| **Te** | PRT - DP | -0.47 | 0.62 | 0.719 |
| **Te** | PRT - FB | 0.51 | 0.30 | 0.205 |
| **EF50** | FB - DP | 168.63 | 106.40 | 0.247 |
| **EF50** | PRT - DP | 141.74 | 124.29 | 0.475 |
| **EF50** | PRT - FB | -26.88 | 59.62 | 0.887 |
| **EIP** | FB - DP | 43.89 | 22.76 | 0.133 |
| **EIP** | PRT - DP | 30.54 | 26.59 | 0.470 |
| **EIP** | PRT - FB | -13.35 | 12.75 | 0.532 |
| **EEP** | FB - DP | -198.35 | 467.56 | 0.899 |
| **EEP** | PRT - DP | 349.76 | 546.19 | 0.786 |
| **EEP** | PRT - FB | 548.11 | 262.01 | 0.096 |
| **TV/BW** | FB - DP | -0.12 | 3.97 | 0.999 |
| **TV/BW** | PRT - DP | 0.50 | 4.63 | 0.993 |
| **TV/BW** | PRT - FB | 0.62 | 2.22 | 0.954 |
| **MV/BW** | FB - DP | 62.55 | 87.30 | 0.741 |
| **MV/BW** | PRT - DP | 43.27 | 101.98 | 0.899 |
| **MV/BW** | PRT - FB | -19.28 | 48.92 | 0.912 |
| **PIF/BW** | FB - DP | -2.68 | 6.64 | 0.908 |
| **PIF/BW** | PRT - DP | -0.44 | 7.76 | 0.998 |
| **PIF/BW** | PRT - FB | 2.24 | 3.72 | 0.808 |
| **PEF/BW** | FB - DP | 10.92 | 7.20 | 0.277 |
| **PEF/BW** | PRT - DP | 11.62 | 8.41 | 0.341 |
| **PEF/BW** | PRT - FB | 0.70 | 4.04 | 0.982 |
| **PEF/PIF** | FB - DP | 0.54 | 0.17 | 0.008 |
| **PEF/PIF** | PRT - DP | 0.49 | 0.20 | 0.042 |
| **PEF/PIF** | PRT - FB | -0.05 | 0.09 | 0.864 |
| **Te/Ti** | FB - DP | -0.74 | 0.28 | 0.030 |
| **Te/Ti** | PRT - DP | -0.38 | 0.33 | 0.480 |
| **Te/Ti** | PRT - FB | 0.37 | 0.16 | 0.061 |
| **PEF/EF50** | FB - DP | -0.12 | 0.19 | 0.797 |
| **PEF/EF50** | PRT - DP | 0.12 | 0.22 | 0.838 |
| **PEF/EF50** | PRT - FB | 0.23 | 0.10 | 0.069 |

**Supplementary Table 3.** Pairwise interbreed comparison between the three breeds (DP: Dobermann Pinscher, FB: French Bulldog, PRT: Parson Russell Terrier), with age included as a covariate. The table reports the estimates, standard errors and the p-values.

| **Parameter** | **Breed Comparison** | **Estimate** | **Standard Error** | **p-value** |
| --- | --- | --- | --- | --- |
| **f** | FB - DP | -0.08 | 2.08 | 0.999 |
| **f** | PRT - DP | -4.59 | 2.01 | 0.071 |
| **f** | PRT - FB | -4.51 | 2.14 | 0.103 |
| **Penh** | FB - DP | -0.15 | 0.32 | 0.885 |
| **Penh** | PRT - DP | 0.33 | 0.31 | 0.532 |
| **Penh** | PRT - FB | 0.48 | 0.33 | 0.316 |
| **PAU** | FB - DP | -0.25 | 0.28 | 0.658 |
| **PAU** | PRT - DP | 0.33 | 0.27 | 0.455 |
| **PAU** | PRT - FB | 0.58 | 0.29 | 0.129 |
| **PIF** | FB - DP | -426.52 | 68.75 | 0.000 |
| **PIF** | PRT - DP | -519.49 | 66.36 | 0.000 |
| **PIF** | PRT - FB | -92.97 | 70.73 | 0.396 |
| **PEF** | FB - DP | -251.91 | 101.11 | 0.045 |
| **PEF** | PRT - DP | -379.90 | 97.59 | 0.001 |
| **PEF** | PRT - FB | -127.99 | 104.02 | 0.443 |
| **Ti** | FB - DP | 0.27 | 0.12 | 0.094 |
| **Ti** | PRT - DP | 0.30 | 0.12 | 0.044 |
| **Ti** | PRT - FB | 0.03 | 0.13 | 0.967 |
| **Te** | FB - DP | -0.13 | 0.27 | 0.878 |
| **Te** | PRT - DP | 0.65 | 0.26 | 0.043 |
| **Te** | PRT - FB | 0.78 | 0.28 | 0.020 |
| **EF50** | FB - DP | -152.68 | 61.98 | 0.048 |
| **EF50** | PRT - DP | -262.60 | 59.83 | 0.000 |
| **EF50** | PRT - FB | -109.92 | 63.76 | 0.210 |
| **EIP** | FB - DP | 25.23 | 11.79 | 0.095 |
| **EIP** | PRT - DP | 7.71 | 11.38 | 0.778 |
| **EIP** | PRT - FB | -17.52 | 12.13 | 0.329 |
| **EEP** | FB - DP | 95.54 | 234.93 | 0.913 |
| **EEP** | PRT - DP | 751.72 | 226.76 | 0.006 |
| **EEP** | PRT - FB | 656.18 | 241.69 | 0.027 |
| **TV/BW** | FB - DP | 1.76 | 2.02 | 0.661 |
| **TV/BW** | PRT - DP | 3.02 | 1.95 | 0.280 |
| **TV/BW** | PRT - FB | 1.26 | 2.08 | 0.817 |
| **MV/BW** | FB - DP | 30.50 | 44.77 | 0.776 |
| **MV/BW** | PRT - DP | 2.59 | 43.21 | 0.998 |
| **MV/BW** | PRT - FB | -27.90 | 46.06 | 0.818 |
| **PIF/BW** | FB - DP | -0.18 | 3.38 | 0.999 |
| **PIF/BW** | PRT - DP | 2.95 | 3.27 | 0.643 |
| **PIF/BW** | PRT - FB | 3.12 | 3.48 | 0.646 |
| **PEF/BW** | FB - DP | 2.93 | 3.75 | 0.717 |
| **PEF/BW** | PRT - DP | 1.51 | 3.62 | 0.908 |
| **PEF/BW** | PRT - FB | -1.42 | 3.86 | 0.929 |
| **PEF/PIF** | FB - DP | 0.15 | 0.09 | 0.226 |
| **PEF/PIF** | PRT - DP | -0.01 | 0.09 | 0.998 |
| **PEF/PIF** | PRT - FB | -0.16 | 0.09 | 0.220 |
| **Te/Ti** | FB - DP | -0.39 | 0.14 | 0.024 |
| **Te/Ti** | PRT - DP | 0.09 | 0.14 | 0.806 |
| **Te/Ti** | PRT - FB | 0.48 | 0.15 | 0.006 |
| **PEF/EF50** | FB - DP | -0.11 | 0.09 | 0.459 |
| **PEF/EF50** | PRT - DP | 0.13 | 0.09 | 0.327 |
| **PEF/EF50** | PRT - FB | 0.24 | 0.10 | 0.041 |
